# NOP2/Sun domain 4-mediated m5C methylation of Janus kinase 1 promotes hepatocellular carcinoma progression

**DOI:** 10.1080/19336918.2026.2757921

**Published:** 2026-11-04

**Authors:** Jingpeng Ke, Yiwen Xu, Fadian Ding, Yiping Chen, Zhimin Li, Jingxi Chen

**Affiliations:** a Department of Hepatobiliary And Pancreatic Vascular Surgery, the First Affiliated Hospital of Xiamen University, School of Medicine, Xiamen University, Xiamen, China; b Department of General Surgery, the First Affiliated Hospital of Xiamen University, School of Medicine, Xiamen University, Xiamen, China; c Department of Hepatopancreatobiliary Surgery, First Affiliated Hospital of Fujian Medical University, Fuzhou, China

**Keywords:** 5-methylcytosine modification, hepatocellular carcinoma, JAK, malignant progression, NSUN4

## Abstract

5-methylcytosine (m5C) modification is associated with various cancers, but the role of NOP2/Sun domain 4 (NSUN4)-mediated m5C modification in hepatocellular carcinoma (HCC) remains unclear. Here, NSUN4 was markedly upregulated in HCC and correlated with poor survival. Silencing NSUN4 suppressed HCC cell proliferation, migration, invasion and tumor growth. Mechanistically, NSUN4 enhanced JAK1 mRNA stability and expression through m5C methylation, activating JAK-STAT3 signaling and driving malignant progression. Thus, NSUN4 promotes HCC progression via m5C-dependent JAK1 stabilization and JAK-STAT3 activation, highlighting NSUN4 as a promising therapeutic target for HCC.

## Introduction

Hepatocellular carcinoma (HCC) is a prevalent malignant tumor globally, representing 75%–85% of all primary liver cancers [1]. For patients with early-stage HCC who undergo surgical intervention, the 5-year survival rate can exceed 70%. However, due to the current lack of efficient early detection methods, most patients are diagnosed at an advanced stage of the disease, which not only makes the prognosis less optimistic but also greatly limits treatment options [2]. In recent years, targeted therapies and checkpoint inhibitors have been utilized in managing advanced HCC [3]. However, clinical studies indicate that these agents exhibit low remission rates and offer limited overall survival benefits [4,5]. Consequently, further exploration into the molecular mechanisms underlying advanced HCC is essential to provide critical insights for the design of more efficacious treatment approaches.

RNA methylation modifications are critical epigenetic modifications in post-transcriptional RNA regulation and are implicated in diverse biological processes [6,7]. 5-Methylcytosine (m5C), a ubiquitous RNA methylation modification, was initially identified in DNA and subsequently discovered to mediate RNA methylation, thereby regulating RNA splicing, nuclear export, stability, and translation [8]. m5C modification is present in a broad range of RNA molecules [9]. The RNA methylation modifications of m5C are regulated by methyltransferases and their binding proteins. At present, the identified m5C RNA methyltransferases mainly include NOP2/SUN RNA methyltransferase family members 1–7 (NSUN1-7) and DNA methyltransferase family (DNMTs) [10]. Numerous studies have shown that m5C modification influences tumor occurrence, maintenance, and progression and may serve as a potential target for cancer therapy. He Y et al. [11] demonstrated that the abundance of m5C in HCC was substantially higher than that in adjacent normal tissues. m5C modifications and their regulators contribute to the development and progression of HCC [12,13]. For example, the m5C RNA methyltransferase NSUN2 mediates the m5C modification of several mRNAs, thereby affecting the cell cycle, metabolism, and sorafenib sensitivity of HCC [14,15]. NSUN4, as a member of the m5C methyltransferase family, has been reported to be abnormally expressed in several cancers, including lung cancer [16], renal cancer [17], and HCC [18]. A previous study by Cui M et al. [19] showed that NSUN4 exhibited significant overexpression in HCC. However, the role and underlying mechanism of NSUN4-driven RNA m5C modification in HCC have yet to be documented.

In this study, we found that NSUN4 was overexpressed in HCC and correlated with the prognosis of patients with HCC. Functional experiments demonstrated that NSUN4 promoted malignant progression in HCC. Furthermore, JAK1 was identified as the target molecule of NSUN4-mediated m5C modification, which can activate phosphorylation of the JAK-STAT3 signaling and further promote the development of malignant phenotypes in HCC. Thus, our findings highlight the effect and regulatory mechanism of NSUN4-mediated m5C modification in HCC.

## Materials and methods

### Clinical samples

Twenty pairs of HCC tissue samples and adjacent non-cancerous liver tissues were collected from the First Affiliated Hospital of Xiamen University between 2021 and 2023. This study was approved by the Ethical Committee of the First Affiliated Hospital of Xiamen University, approval number: [2021]KEYANLUNSHENZI(013)HAO. Written informed consent was obtained from each participant.

### Cell culture

HepG2 and Hep3B cells were cultured in DMEM medium, while HCCLM3, Huh7, and MIHA cells were cultured in RPMI 1640 medium.

### Plasmids and transfection

NSUN4 siRNAs were provided by GenePharma (Shanghai, China). The transfection of these siRNAs was carried out using Lipo3000 (Thermo Fisher Scientific, USA). JAK1 cDNA was subcloned into the pcDNA3.1 expression vector. Plasmid transfection was carried out with Lipofectamine 2000 (Thermo Fisher Scientific, USA).

### Western blotting

Western blotting was carried out as previously reported [20]. The antibodies utilized in this research are detailed below: anti-NSUN4 (Abcam, ab235430), anti-JAK1 (Solarbio, K001410M), anti-STAT3 (Proteintech, 60,199–1-Ig), anti-p-STAT3 (MCE, HY-P80857), anti-β-actin (Proteintech, 66,009–1-Ig), and anti-GAPDH (Proteintech, 60,004–1-Ig).

### Colony formation, wound-healing, and transwell invasion assays

Colony formation, Wound-healing, and Transwell invasion assays were conducted as described previously [12].

### RNA stability assay

HCCLM3 and Huh-7 cells were plated in 6-well plates and subsequently exposed to Actinomycin D (5 µg/mL, MedChemExpress) for durations of 0, 30, 60, and 90 min. Then, cells were collected for RNA isolation. The mRNA levels of JAK1 were measured using qRT-PCR.

### RNA immunoprecipitation (RIP)

Cells were incubated with RIP lysis buffer for 30 min. A portion of the cell lysate, specifically 1/10 of the total volume, was reserved as input. The remaining cell lysates were incubated with either anti-m5C-coated beads (anti-IgG as negative control) for 4 h. Subsequently, the complex was washed. Then, the immunoprecipitated samples were digested with proteinase K, and the precipitated RNA was obtained. The enrichment of JAK1 mRNA was detected using qRT-PCR.

### Animal studies

Fifteen male BALB/c nude mice (6–8 weeks old) were obtained from Shanghai Slaccas Laboratory Animal Co., Ltd. (Shanghai, China). These mice were randomly assigned to three groups: negative control (NC), LV-sh-NSUN4+vector, and LV-sh-NSUN4+JAK1, 6 mice/per group. HCC-LM3 cells (5.0 × 10^6^) transduced with the corresponding plasmids were injected subcutaneously into the right flank of the mice. The width (W) and length (L) of the tumor were recorded every 7 days, and tumor volume was calculated using the formula V=(L×W^2^)/2. At the 4th week after injection, the mice were anesthetized by intraperitoneal injection of pentobarbital sodium (60 mg/kg) and euthanized by cervical dislocation. Tumors were then harvested for further analysis. This study was approved by the Animal Experimental Ethics Committee of Fujian Medical University (No: IACUC FJMU 2024–0150).

### Statistical analysis

Data were presented as mean ± standard error of the mean. Student’s t-tests and one-way analysis of variance (ANOVA) were applied to assess differences between groups. Pearson’s correlation coefficient method was utilized to examine expression correlation. Statistical significance was determined when the *p* value was less than 0.05.

## Results

### NSUN4 is upregulated in HCC and related to prognosis in HCC patients

Using the University of Alabama at Birmingham Cancer Data Analysis Portal (UALCAN), it was revealed that NSUN4 mRNA expression exhibited significant upregulation in HCC (Figure 1(A)). Meanwhile, the expression levels of NSUN4 mRNA were confirmed to be upregulated in HCC tissues compared with adjacent normal tissues (Figure 1(B)). Moreover, analysis of data from the Clinical Proteomic Tumor Analysis Consortium (CPTAC) database revealed that NSUN4 protein levels were significantly elevated in HCC (Figure 1(C)). Similarly, Western blot analysis and immunohistochemical (IHC) validated that the expression of NSUN4 protein in 20 pairs of HCC tissue was up-regulated compared with adjacent normal tissue (Figure 1(D,E)). Furthermore, the protein level of NSUN4 was more abundant in four HCC cell lines than in normal hepatocytes MIHA (Figure 1(F)). In addition, Kaplan–Meier analysis from the Gene Expression Profiling Interactive Analysis (GEPIA) database revealed that HCC patients with higher *NSUN4* expression had shorter overall survival (Figure 1(G)).

**Figure 1.**
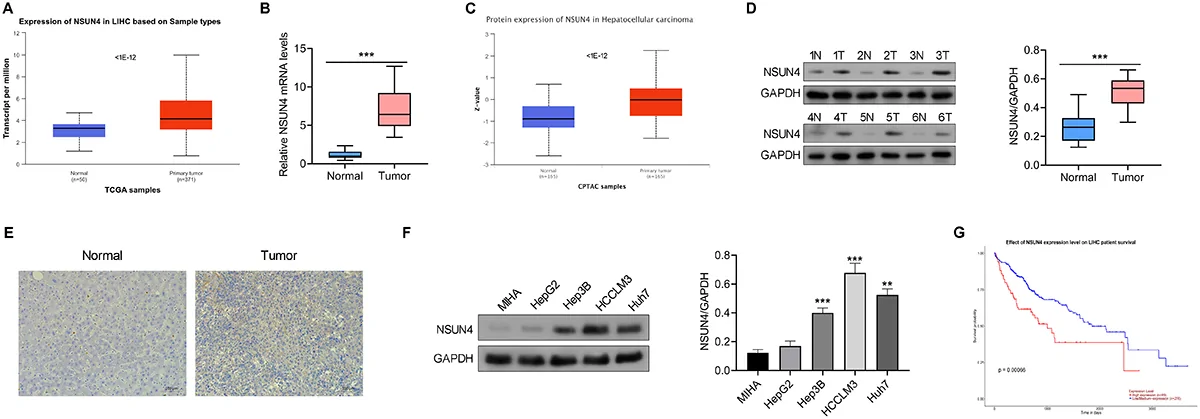
NSUN4 is upregulated in HCC, associated with patient survival. (A) NSUN4 was highly expressed in HCC tumor tissues compared with adjacent normal tissues from the UALCAN database. (B) qRT‑PCR analysis showed the NSUN4 mRNA levels in 20 paired samples of HCC tumors (T) and corresponding adjacent normal tissues (N). (C) CPTAC database showed that the protein level of NSUN4 was remarkably increased in HCC. (D) Western blot analysis showed the NSUN4 protein levels in paired samples of HCC tumors and corresponding adjacent normal tissues. (E) Immunohistochemical staining of NSUN4 in HCC tumors and corresponding adjacent normal tissues. (F) Western blot analysis of NSUN4 in HCC cell lines (HepG2, Hep3B, HCCLM3, and Huh7) and normal hepatocytes MIHA. (G) Kaplan–Meier estimates of survival time of patients in cohort (*n* = 365) from UALCAN by different NSUN4 expression levels in tumors. **p < .01; ***p < .001.

### NSUN4 enhances the malignant potential of HCC cells

The role of NSUN4 in HCC was studied by transfecting NSUN4 siRNAs into HCCLM3 and Huh-7 cells. As shown in Figure 2(A), out of the three designed and synthesized siRNAs, si-NSUN4#1 had the most significant inhibitory effect on NSUN4 expression in HCC cells and was therefore selected for subsequent experiments. The Cell Counting Kit-8 (CCK-8) assay results showed that knocking down NSUN4 reduced the proliferation of HCCLM3 and Huh-7 cells (Figure 2(B)). Similarly, colony formation assays revealed that knockdown of *NSUN4* inhibited anchorage-independent growth, as evidenced by a decrease in colony numbers (Figure 2(C)). We further examined the impact of *NSUN4* on migratory and invasive potential using wound‑healing and transwell assays. The results showed that silencing *NSUN4* suppressed migratory and invasive potential (Figure 2(D,E)). Moreover, flow cytometry analysis revealed that the knockdown of NSUN4 considerably enhanced cell apoptosis (Figure 2(F)).

**Figure 2.**
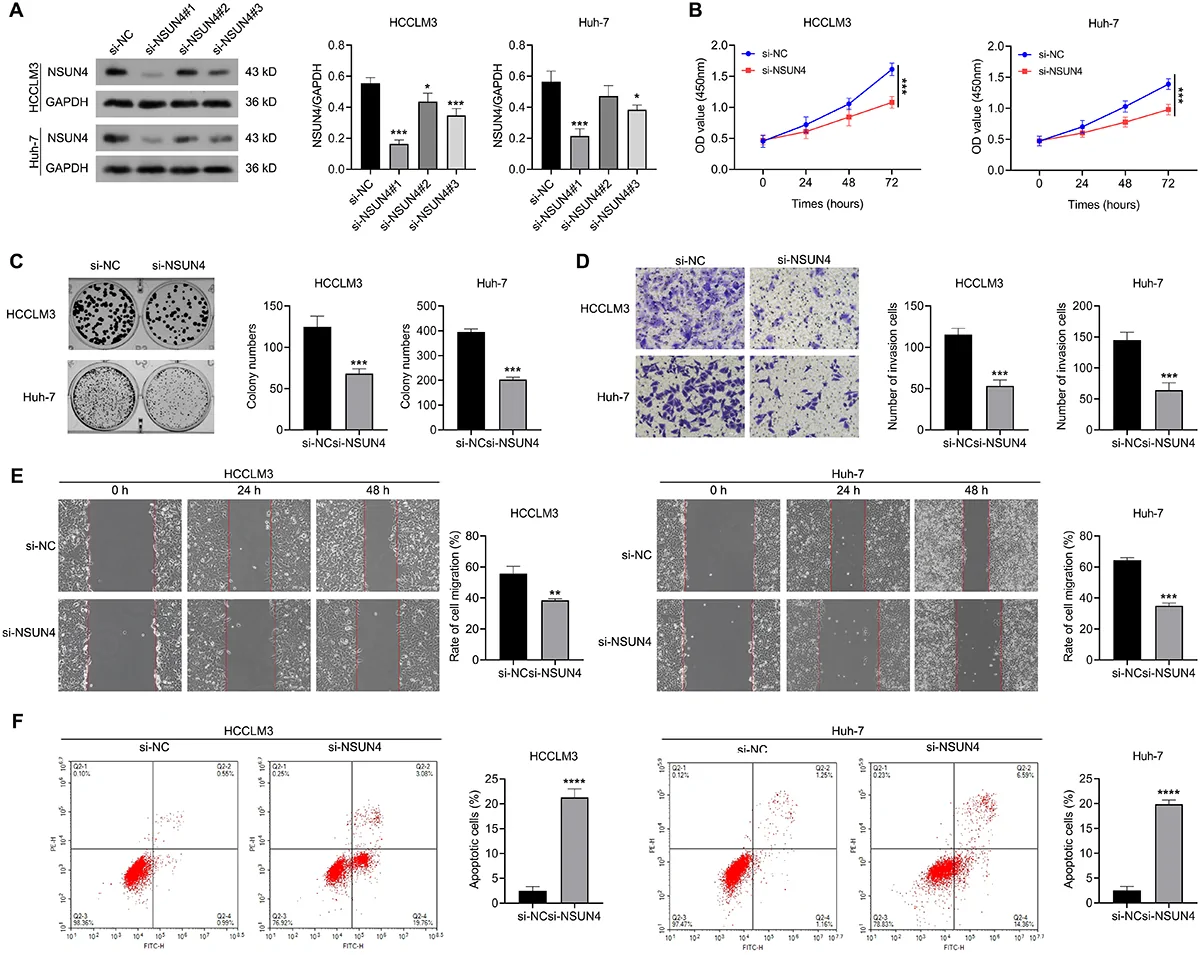
NSUN4 promotes malignant phenotypes of HCC cells. (A) After infection of HCCLM3 and Huh-7 cells with siRNAs, the expression of NSUN4 was analyzed by Western blot. (B) Silencing NSUN4 inhibited HCC cell proliferation, determined by CCK-8 assays. (C) Knockdown of NSUN4 suppressed the clonogenic ability of HCCLM3 and Huh-7 cells. (D) After transfection of HCCLM3 and Huh-7 cells with siRNAs, cell invasion was determined in NSUN4-silenced cells by transwell assays. Scale bar = 100 µm. (E) Cell migration was determined by wound‑healing assays. Scale bar = 200 µm. (F) Flow cytometry assay of cell apoptosis. *p < .05,**p < .01; ***p < .001.

### NSUN4 regulates JAK1 mRNA m5C methylation and then enhances JAK1 mRNA stability and translation

Therefore, how *NSUN4* affects malignant phenotypes of HCC cells is an interesting scientific question worthy of further exploration. We analyzed the genes correlated with NSUN4 in the LIHC database. KEGG pathway analysis revealed that the genes positively correlated with NSUN4 were mainly involved in chemical carcinogenesis-receptor activation, pathways in cancer, and the PI3K-Akt signaling pathway (Figure 3(A)), whereas the genes negatively correlated with NSUN4 were enriched in cholesterol metabolism and the PPAR signaling pathway (Figure 3(B)). JAK1, a critical factor in the oncogenic JAK-STAT pathway, was positively correlated with NSUN4 (Pearson correlation coefficient: 0.73, Figure 3(C)). The UALCAN database showed that the levels of JAK1 were markedly increased in HCC (Figure 3(D)). Moreover, depletion of *NSUN4* led to a marked decrease in both the mRNA and protein expression levels of JAK1 (Figure 3(E,F)). In addition, the depletion of *NSUN4* remarkably inhibited the phosphorylation level of STAT3, the downstream protein of JAK1 (Figure 3(G)).

**Figure 3.**
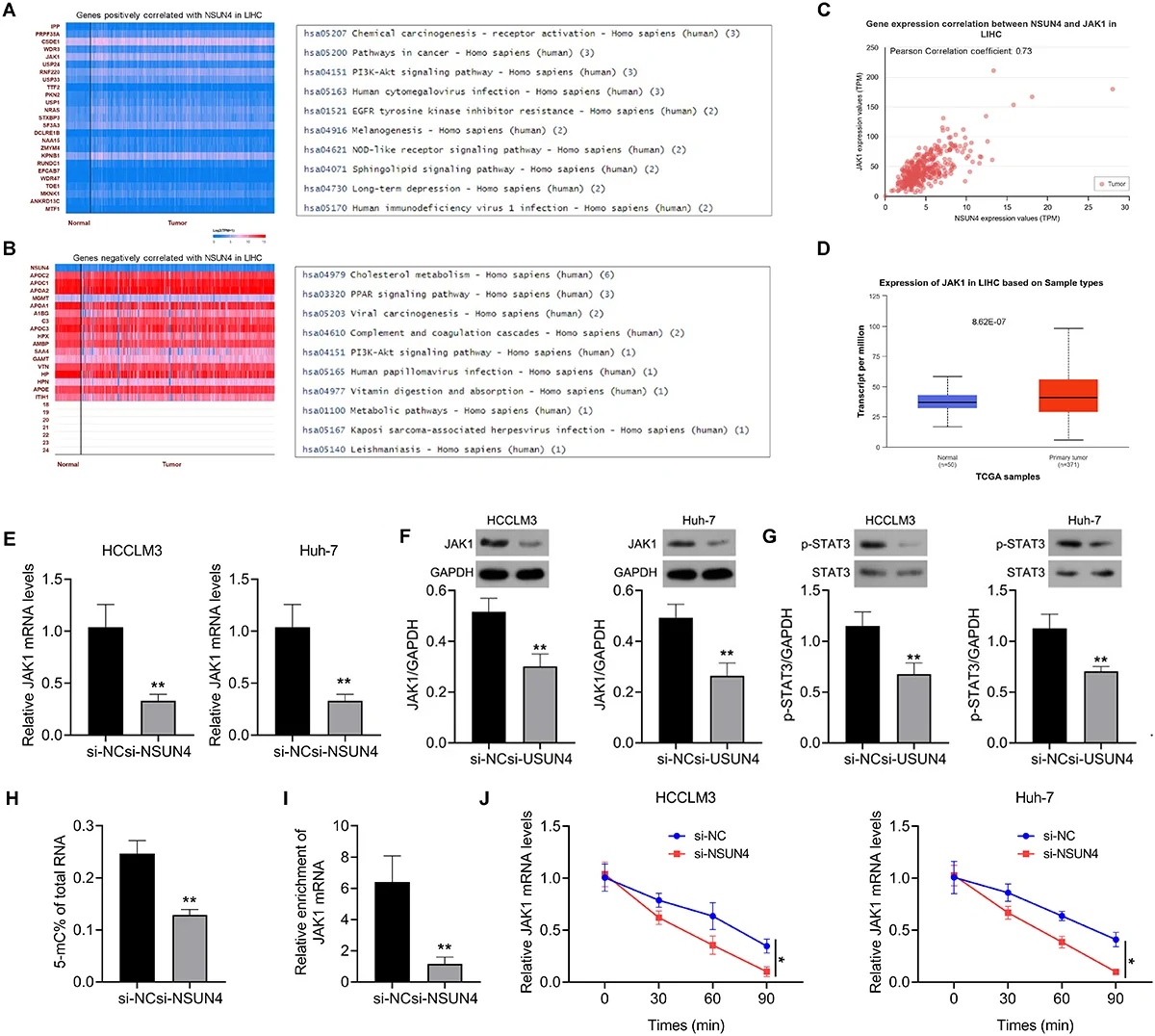
NSUN4 regulates JAK1 mRNA m5C methylation and then enhances JAK1 mRNA stability and translation. (A) KEGG enrichment analysis of genes positively correlated with NSUN4 in HCC. (B) KEGG enrichment analysis of genes negatively correlated with NSUN4 in HCC. (C) Correlation between NSUN4 and JAK1. (D) JAK1 was highly expressed in HCC tumor tissues compared with adjacent normal tissues from the UALCAN database. (E) qRT‑PCR analysis of JAK1 mRNA in NSUN4-knockdown HCC cells. (F) Western blot analysis of JAK1 protein expression in NSUN4-knockdown HCC cells. (G) The effect of NSUN4 knockdown on STAT3 in HCC cells, measured via Western blotting. (H) m5C-RIP followed by qRT‑PCR confirmed that JAK1 mRNA was enriched by the m5C antibody. (I) m5C-RIP followed by qRT-PCR in NSUN4-deficient HCCLM3 cells indicated that NSUN4 mediated the m5C modification of JAK1 mRNA. (J) mRNA stability of *JAK1* was evaluated in NSUN4-deficient HCC cells. *p < .05, **p < .01, ***p < .001.

NSUN4, as an RNA methyltransferase, catalyzes the methylation of cytosine to 5-methylcytosine, thereby regulating RNA stabilization and expression [21]. Thus, we speculate that NSUN4 may affect the expression of JAK1 mRNA through m5C modification. We found that *JAK1* mRNA was effectively enriched by the m5C antibody (Figure 3(H)). Furthermore, silencing NSUN4 reduced the m5C levels of *JAK1* in HCC cells (Figure 3(I)). Since m5C modification can regulate target mRNA stability, to determine how NSUN4-mediated m5C regulates the expression of JAK1 mRNA, we treated HCC cells with actinomycin D. As illustrated in Figure 3(J), the silencing of NSUN4 markedly decreased the half-life of JAK1 mRNA. These results indicate that NSUN4 upregulates JAK1 by enhancing its mRNA stability via m5C modification.

### JAK1 is essential for the NSUN4-mediated malignant potential of HCC cells

JAK1-overexpressing plasmids were constructed, and Western blot assay in HCCLM3 and Huh-7 cells confirmed overexpression of JAK1 (Figure 4(A)). To investigate the implication of JAK1 in the NSUN4-mediated malignant potential of HCC cells, rescue assays were performed in NSUN4-deficient HCC cells. CCK-8 and colony formation assays indicated reduced proliferation in NSUN4-knockdown cells, mitigated by forced expression of JAK1 (Figure 4(B,C)). Furthermore, wound‑healing and transwell experiments in HCCLM3 and Huh-7 cells demonstrated that NSUN4 knockdown resulted in decreased cell migration and invasion, which could be counteracted by *JAK1* overexpression (Figure 4(D,E)). Flow cytometry assay showed that JAK1 overexpression significantly reversed the NSUN4 knockdown-induced increase in apoptosis (Figure 4(F)), consistent with restoration of JAK1–STAT3-mediated pro-survival signaling. These results suggest that NSUN4 promotes HCC cell malignant potential through upregulating JAK1 expression.

**Figure 4.**
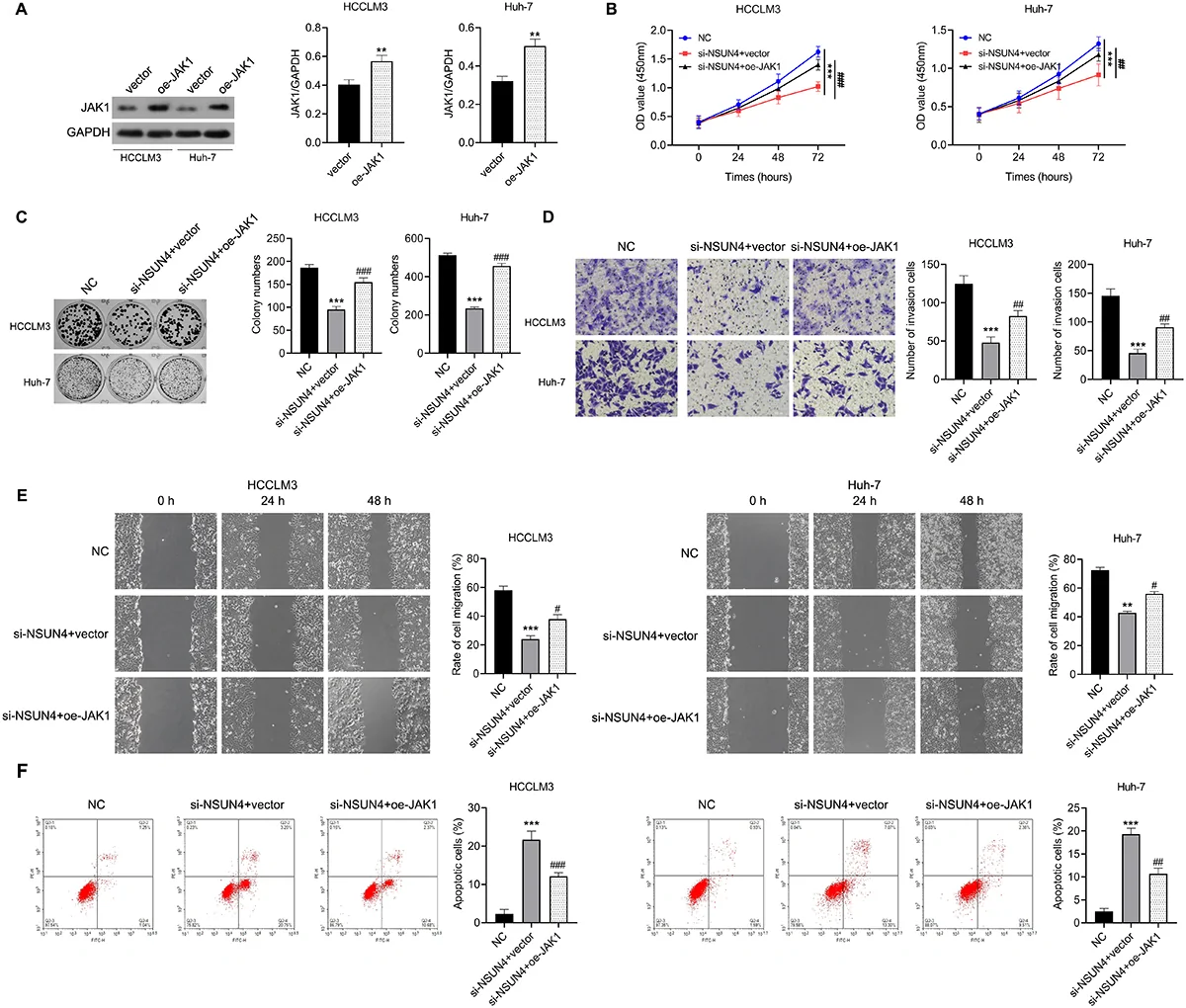
JAK1 is crucial for the NSUN4-mediated malignant potential of HCC cells. (A) Western blot demonstrating JAK1 expression in HCC cells transfected with JAK1-overexpressing plasmids. **p < .01 compared with the vector group. (B) CCK-8 assay. (C) Colony formation assay. (D) Wound healing. (E) Transwell invasion assay. (F) Flow cytometry assay of cell apoptosis. **p < .01, ***p < .001 versus the NC group; # *p* < .05, ## *p* < .01, ### *p* < .001 versus the sh-NSUN4+vector group.

### NSUN4 enhances HCC growth by activating the JAK1-STAT3 signaling pathway

The oncogenic role of NSUN4 in HCC was investigated in vivo by constructing a subcutaneous tumorigenesis model. We established three experimental groups: NC, LV-sh-NSUN4+vector, and LV-sh-NSUN4+oe-JAK1 in HCCLM3 cells (Figure 5(A)). As presented in Figure 5(B,C), both the volume and weight of the NSUN4-knockdown group were markedly reduced compared with those of the NC group, while overexpression of *JAK1* reversed the suppressive effects of NSUN4 knockdown. Moreover, IHC staining demonstrated that tumors in the NSUN4-knockdown group displayed reduced cellular proliferation, whereas overexpression of JAK1 restored this effect (Figure 5(D)). Furthermore, NSUN4 knockdown inhibited the expression of JAK1 and p-STAT3, and overexpression of JAK1 re-activated the JAK1-STAT3 signaling pathway (Figure 5(E)). Collectively, these results support the hypothesis that NSUN4 promotes the malignant progression of HCC by activating the JAK1-STAT3 signaling pathway.

**Figure 5.**
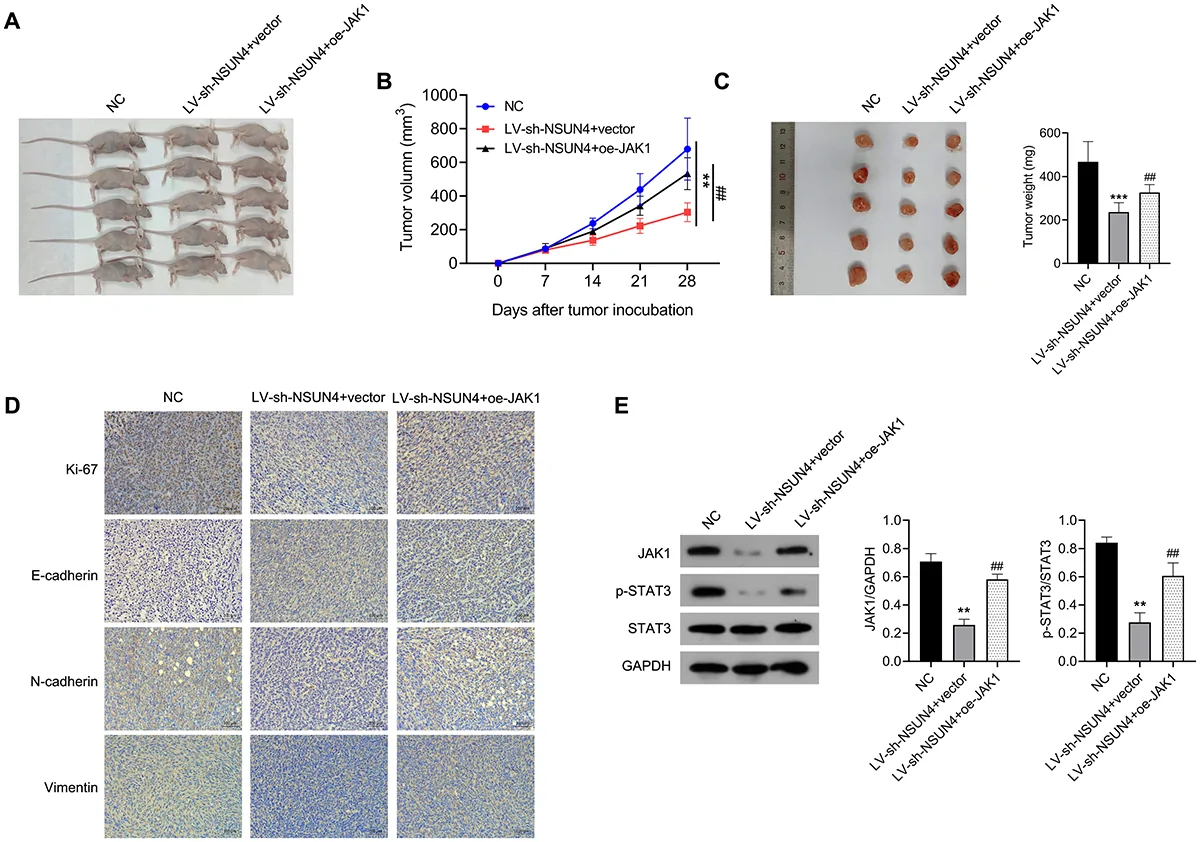
NSUN4 promotes tumor growth of HCC by activating the JAK1-STAT3 signaling pathway. (A) Three experimental groups: NC, LV-sh-NSUN4+vector, and LV-sh-NSUN4+oe-JAK1. Transfected HCCLM3 cells were subcutaneously injected into BALB/c nude mice. (B) The volume of the tumors was measured every week. (C) Tumor weight. (D) Representative IHC images of Ki‑67, E-cadherin, N-cadherin, and vimentin in xenograft tumors. Scale bar = 100 µm. (E) Western blotting analysis of JAK1, p-STAT3, and STAT3 in xenograft tumors. **p < .01, ***p < .001 compared with the NC group; ## *p* < .01 compared with the LV-sh-NSUN4+vector group.

## Discussion

HCC is a highly lethal malignancy with significant global implications, accounting for over 40% of new cases and deaths occurring in China. Of particular concern is the unsatisfactory therapeutic approach currently available for patients with advanced or recurrent HCC. In this study, we demonstrate that NSUN4 is markedly upregulated in HCC and correlates with an unfavorable prognosis. Downregulation of NSUN4 expression effectively impairs the proliferation, migration, and invasion of HCC cells, as well as tumor growth *in vivo*. Mechanistically, elevated NSUN4 expression leads to increased m5C modification of JAK1 mRNA, resulting in enhanced stability. These findings indicate that NSUN4 may represent a promising therapeutic target for HCC.

Post-transcriptional RNA modifications are widely involved in tumorigenesis and tumor progression [22,23]. m5C, an RNA modification, dynamically modulates a range of biological functions [24]. Several m5C regulatory factors have been found to play critical roles in the pathogenesis and progression of HCC [14,25,26]. It has been reported that NSUN4 expression is increased in HCC, and its high expression is linked to an unfavorable prognosis [19]. Consistent with these reports, in a cohort of 20 pairs of HCC tissues and adjacent normal tissues, we found that NSUN4 is highly expressed in HCC at the transcriptional and translational levels. Subsequently, our study has elucidated the critical role of NSUN4 in modulating malignant phenotypes in HCC. Depletion of NSUN4 inhibited the malignant growth of HCC in vitro and in vivo. Our findings demonstrate the potential beneficial effect of targeting NSUN4 in HCC treatment and suggest the possibility of developing anti-HCC therapeutic strategies targeting NSUN4 in the future.

m5C-methyltransferase NSUN4 affects the stability of target RNA via mediating m5C modification. NSUN4 was initially identified as a mitochondrial rRNA methyltransferase. Metodiev MD and colleagues [21] demonstrated that NSUN4 facilitates the methylation of the 12S rRNA within the small ribosomal subunit in mitochondria, thereby promoting rRNA rearrangements to establish a peptidyl transferase center. Recently, a study revealed the m5C modification of NSUN4 on mRNA. Yang L et al. [27] demonstrated that NSUN4 regulated m5C methylation and translational reprogramming of Sox9 by binding to the 3’UTR, thereby promoting chondrogenic differentiation. In our study, JAK1 was predicted to be a target gene of NSUN4 by bioinformatic analysis. JAK1 is known as a critical factor in the JAK-STAT pathway, which is an important oncogenic pathway in numerous cancers, including HCC [28,29]. Yang L et al. [30] reported a significant upregulation of JAK1 mRNA expression in HCC specimens compared with controls. The use of a JAK1 inhibitor resulted in reduced growth of HepG2 and Huh-7 cells. In this study, we confirmed that NSUN4 is capable of catalyzing the m5C modification of JAK1, thereby regulating its expression and facilitating HCC progression. Additional functional rescue experiments clarified the link between NSUN4-mediated m5C modification of JAK1 mRNA and the development of HCC. Our findings also indicated that the JAK1-STAT3 pathway was activated in HCC cells, while silencing of NSUN4 led to deactivation of this pathway.

Mechanistically, the apoptotic phenotype observed upon NSUN4 manipulation can be explained by the JAK1–STAT3 survival axis. JAK1 phosphorylates STAT3, and activated STAT3 sustains a pro-survival transcriptional program (MCL1, BCL-xL, survivin, and MYC) while repressing pro-apoptotic FAS, thereby raising the apoptotic threshold [31,32]. Constitutive STAT3 activation is also a recognized determinant of the anti-apoptotic phenotype in HCC [33]. Our findings suggest that NSUN4-mediated m5C stabilization of JAK1 sustains p-STAT3 and this survival program. Accordingly, loss of NSUN4 weakens STAT3-dependent survival signaling and shifts the balance toward apoptosis, whereas restoring JAK1 reengages STAT3 and reduces apoptosis, supporting that this apoptotic phenotype is JAK1-dependent. Future studies should examine BCL-2 family members, caspase cleavage, and mitochondrial function to test this model.

## Conclusions

This study elucidates a potential mechanism through which NSUN4 facilitates HCC progression by augmenting JAK1 mRNA stabilization and expression in an m5C-dependent manner. These findings provide mechanistic insight into the oncogenic role of NSUN4-mediated m5C modification in HCC, highlighting the significance of NSUN4 as a promising therapeutic target for HCC treatment.

## Abbreviations

HCC: Hepatocellular carcinoma
m5C: 5-Methylcytosine
UALCAN: University of Alabama at Birmingham Cancer Data Analysis Portal
CPTAC: Clinical Proteomic Tumor Analysis Consortium
GEPIA: Gene Expression Profiling Interactive Analysis
KEGG: Kyoto Encyclopedia of Genes and Genomes
TCGA-LIHC: The Cancer Genome Atlas Liver Hepatocellular Carcinoma
rRNAs: ribosomal RNAs
tRNAs: transfer RNAs
mRNAs: message RNAs
NSUN1-7: NOP2/SUN RNA methyltransferase family members 1–7
DNMTs: DNA methyltransferase family
JAK1: Janus kinase 1
qRT-PCR: quantitative real-time PCR
RIP: RNA immunoprecipitation
CCK-8: Cell Counting Kit-8
IHC: Immunohistochemistry
SEM: standard error of the mean
ANOVA: Students’ t-tests and analysis of variance
